# Ocular Adverse Effects of Intravitreal Bevacizumab Are Potentiated by Intermittent Hypoxia in a Rat Model of Oxygen-Induced Retinopathy

**DOI:** 10.1155/2017/4353129

**Published:** 2017-07-09

**Authors:** Jeffrey J. Tan, Charles L. Cai, Eric M. Shrier, Lois McNally, Douglas R. Lazzaro, Jacob V. Aranda, Kay D. Beharry

**Affiliations:** ^1^Department of Ophthalmology, State University of New York, Downstate Medical Center, Brooklyn, NY, USA; ^2^State University of New York Eye Institute, New York, NY, USA; ^3^Department of Pediatrics, Division of Neonatal-Perinatal Medicine, State University of New York, Downstate Medical Center, Brooklyn, NY, USA

## Abstract

Intravitreal bevacizumab (Avastin) use in preterm infants with retinopathy of prematurity is associated with severe neurological disabilities, suggesting vascular leakage. We examined the hypothesis that intermittent hypoxia (IH) potentiates intravitreal Avastin leakage. Neonatal rats at birth were exposed to IH from birth (P0)–P14. At P14, the time of eye opening in rats, a single dose of Avastin (0.125 mg) was injected intravitreally into the left eye. Animals were placed in room air (RA) until P23 or P45 for recovery (IHR). Hyperoxia-exposed and RA littermates served as oxygen controls, and equivalent volume saline served as the placebo controls. At P23 and P45 ocular angiogenesis, retinal pathology and ocular and systemic biomarkers of angiogenesis were examined. Retinal flatmounts showed poor peripheral vascularization in Avastin-treated and fellow eyes at P23, with numerous punctate hemorrhages and dilated, tortuous vessels with anastomoses at P45 in the rats exposed to IH. These adverse effects were associated with robust increases in systemic VEGF and in both treated and untreated fellow eyes. Histological analysis showed severe damage in the inner plexiform and inner nuclear layers. Exposure of IH/IHR-induced injured retinal microvasculature to anti-VEGF substances can result in vascular leakage and adverse effects in the developing neonate.

## 1. Introduction

Retinopathy of prematurity (ROP) has been extensively studied in clinical trials but remains a major cause of severe irreversible blindness worldwide. The exact physiologic mechanisms and optimal treatment of significant clinical disease are poorly elucidated. Extremely low gestational age neonates (ELGANs) requiring excessive supplemental oxygen, and who experience frequent arterial oxygen desaturations or apneas, are at a high risk for pathologic retinal vasculature development [1–3]. This process of “threshold” retinopathy ultimately leads to subnormal anatomic and functional development: permanently underdeveloped retina, macular dragging, retinal detachment, neovascular glaucoma, blindness, and ultimately phthisis bulbi [4]. The incidence of ROP in developed countries is estimated to be 5–8% of preterm infants while in developing countries, it may be as high as 30% [5]. Despite advancements in treatment, the number of individuals with blindness due to ROP is still as high as 50,000 worldwide [6].

Although long-term studies such as Cryotherapy for Retinopathy of Prematurity (CRYO-ROP) showed reduction of disease compared to observation, almost half (44.7%) of cryotherapy-treated eyes still had unfavorable visual acuity outcomes [4]. Laser panretinal photocoagulation has surpassed cryotherapy as the standard of care, as the Early Treatment in Retinopathy of Prematurity Study (ETROP) showed at least equal success to cryotherapy in all treated eyes and significantly improved outcomes in those with the most posterior and severe disease [7]. Laser photocoagulation ablates the undeveloped retina, does not allow for full vascularization, and potentially exaggerates myopia [8]. On the other hand, its effects are durable and permanent. A myriad of trials have sought to parse out the associated exacerbating and remitting factors to allow improved success with treatment, especially with aggressive posterior ROP (APROP) where functional and anatomic success is low [9–11]. Of the newer treatment modalities, off-label use of intravitreal bevacizumab (Avastin), a recombinant humanized vascular endothelial growth factor inhibitor (anti-VEGF) that binds to all VEGF-A isoforms, has shown the most promise as it does not immediately destroy any retinal tissue as does laser therapy. The landmark BEAT-ROP study demonstrated advantage of intravitreal bevacizumab over laser therapy for zone 1 stage 3+ ROP by improving structural outcomes, decreasing recurrence, and allowing continued development of peripheral retina [12, 13]. However, its use in ROP is somewhat controversial. In the BEAT-ROP study, assessment of local and systemic safety profile could not be determined due to study size [12]. With Avastin, it is also the case that significant, potentially blinding ROP may develop at an altered time course, making close and extended follow-up critical [14]. The occurrence of APROP coincides with crucial stages of development of vital organs such as the brain, heart, lungs, and kidneys. These structures require VEGF for normal development, and any alterations caused by anti-VEGF therapies may cause long-term adverse effects [15]. Avastin is a highly potent anticancer agent, used off-label for ophthalmic conditions, and is not formulated for use within the eye. Recent studies have since tried to address safety of intravitreal Avastin in ROP and demonstrated numerous acute and latent retinal adverse effects [16–20], as well as a higher incidence of severe neurological disabilities [21].

Avastin is considered as a species-specific antibody for use in humans. However, numerous studies have reported Avastin efficacy in rats [22–28]. Avastin was shown to bind murine VEGF in three independent molecular bioassays [24]. Furthermore, toxicology studies conducted in rabbits showed reduced wound healing, decreased maternal and fetal body weights, increased fetal resorptions, and a number of teratogenic effects (Avastin package insert), demonstrating that Avastin effects are not exclusive to humans. Therefore, using an established rat model of oxygen-induced retinopathy (OIR), which closely simulates the frequent, brief intermittent hypoxic (IH) episodes (or apneas) experienced by ELGANs, we tested the hypothesis that the numerous adverse effects of Avastin that are reported in the literature may be due to potentiation by IH. The rationales for this hypothesis were (a) ELGANs experience several hundred episodes of IH during the first weeks of postnatal life, (b) IH causes endothelial impairment and function leading to vascular leakiness, (c) restitution of blood flow through severely injured microvasculature during reoxygenation following an IH episode (IHR) leads to hemorrhage, and (d) leakage into the systemic circulation will result in adverse effects in the developing neonate.

## 2. Materials and Methods

### 2.1. Experimental Design

All experiments were approved by the State University of New York (SUNY), Downstate Medical Center Institutional Animal Care and Use Committee, Brooklyn, NY. Certified infection-free, timed-pregnant Sprague-Dawley rats were purchased from Charles River Laboratories (Wilmington, MA) at 17 days gestation. The animals were housed in an animal facility with a 12-hour day/12-hour night cycle and provided standard laboratory diet and water ad libitum until delivery. Within 2-3 hours of birth, newborn rat pups delivering on the same day were pooled and randomly assigned to expanded litters of 18 pups/litter (9 males and 9 females). Gender was determined by the anogenital distance. The expanded litter size was used to simulate relative postnatal malnutrition of extremely low gestational age newborns (ELGANs) who are at increased risk for severe ROP. Each pup was weighed and measured for linear growth (crown to rump length in centimeters) and randomized to either (1) room air (RA), (2) hyperoxia (50% O_2_), or (3) intermittent hypoxia (IH, 50/12% O_2_ cycling) from P0 to P14. The IH cycling profile consisted of hyperoxia (50% O_2_) with brief (1 minute) hypoxia (12% O_2_) episodes (3 clusters, 10 minutes apart) for a total of 8 clustered hypoxic episodes per day. This clustering design has been shown to produce a severe form of oxygen-induced retinopathy (OIR) in neonatal rats [29–34] and confirmed in human neonates [3]. At P14 (time of eye opening in rats), animals were anesthetized with halothane/oxygen, povidone iodine was placed on the conjunctiva, and 5 *μ*L of Avastin was injected into the left eye. A volume and dose of Avastin were based on previous studies [23]. Preterm infants receive 0.625 mg Avastin per eye for a total concentration of 1.25 mg. We injected only one eye with a total concentration of 0.125 mg. The right eyes received equivalent volume sterile normal saline and served as placebo controls. Sterile normal saline was used as the placebo control according to the manufacturer's instructions in the package insert to dilute Avastin in 0.9% sodium chloride. Other investigators have used intravitreal balanced salt csolution as placebo controls [23]. The animals were monitored daily for signs of infection. At P23 and P45, the animals were euthanized and blood and eyes collected for the assessment of VEGF, sVEGFR-1, and IGF-I; retinal angiogenesis and pathology; and expression of ocular angiogenesis biomarkers.

### 2.2. Sample Collection

Both eyes from 9 male and 9 female pups in each group were enucleated and rinsed in ice-cold phosphate-buffered saline (pH 7.4) on ice. The vitreous fluid (VF) samples were aspirated as previously described [29, 31–34] using a sterile 27-gauge needle attached to a 0.5 mL tuberculin syringe. VF samples were pooled to obtain 3 male and 3 female samples in each group. The retinas and choroids were then excised and processed as previously described [29, 31–34]. To obtain enough tissue, samples were pooled and a total of 6 samples (3 males and 3 females) per group were analyzed. Pooled samples were placed in sterile individual tubes containing ceramic beads and homogenized using a Fast-Prep 24 system (MP Biomedicals, Solon, OH, USA) in 1.0 mL ice-cold sterile normal saline. The homogenates were centrifuged at 10,000 rpm for 20 minutes at 4°C and filtered prior to assay. A portion of the filtrate (10 *μ*L) was used for total cellular protein levels. For collection of blood samples, the rat pups were euthanatized by decapitation. Mixed arterial-venous blood samples were collected in sterile Eppendorf tubes and placed on ice for 30 minutes, prior to centrifugation at 3000 rpm at 4°C.

### 2.3. Assay of VEGF, sVEGFR-1, and IGF-I

VEGF, sVEGFR-1, and IGF-I levels were determined in the serum and retinal and choroidal homogenates using commercially available rat sandwich immunoassay kits (R & D Systems, Minneapolis, MN, USA) according to the manufacturer's protocol. Data from the homogenates were standardized using total cellular protein levels as previously described [29, 31–34]. Validation studies comparing serum and plasma VEGF levels show no differences [35]. In addition, recovery studies done by the manufacturer, of human VEGF spiked to three different levels throughout the range of the assay in various matrices showed 102% recovery for serum (range: 92–115%, *n* = 5); 97% recovery for EDTA plasma (range: 82–113%, *n* = 5); 93% recovery for heparin plasma (range: 82–102%, *n* = 5); and 100% recovery for citrate plasma (range: 88–113%, *n* = 5), providing further evidence that either plasma or serum can be used to determine VEGF levels with equivalent validity.

### 2.4. Total Cellular Protein Assay

On the day of the assay, retinal homogenates were assayed for total protein levels using the dye-binding Bio-Rad protein assay (Bio-Rad, Hercules, CA, USA) with bovine serum albumin as a standard.

### 2.5. ADPase Staining

ADPase staining of the retinas and computer imaging were carried out as previously described [29, 31–34].

### 2.6. Retinal H&E Staining

Eyes were enucleated, rinsed in phosphate-buffered saline (PBS), fixed in Hartmann's fixative, and sent to New York University Experimental Pathology Histology Core Laboratory, NY, NY, USA, for processing and staining using standard histological techniques. Images were captured at 40x magnification using an Olympus BX53 microscope, DP72 digital camera, and CellSens imaging software (Olympus, Center Valley, PA, USA), attached to a Dell Precision T3500 computer (Dell, Round Rock, TX, USA).

### 2.7. Vascular Density Quantification

**Figure 1:**
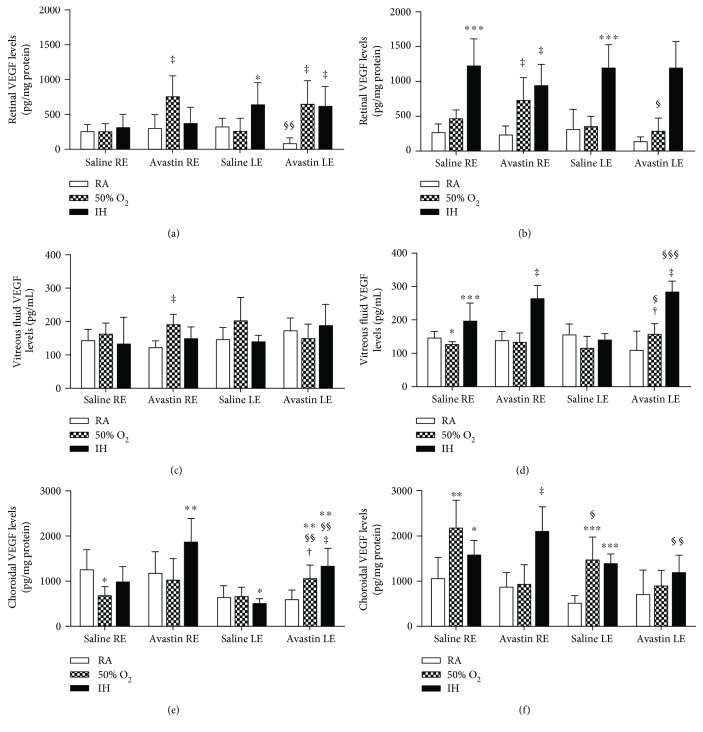
Effects of Avastin on retinal (a, b), vitreous fluid (c, d), and choroidal (e, f) VEGF levels in adolescent 23-day-old (a, c, and e) and pubertal 45-day-old (b, d, and f) rats exposed to room air (RA), hyperoxia (50% O_2_), and intermittent hypoxia (IH). Animals exposed to IH received 50% O_2_ with brief, clustered hypoxia (12% O_2_) episodes for a total of 8 episodes per day from P0 to P14. Data are presented as mean ± SD (*n* = 6 samples/group). ^∗^*p* < 0.05, ^∗∗^*p* < 0.01, ^∗∗∗^*p* < 0.001 versus saline RA; ^†^*p* < 0.05, ^‡^*p* < 0.01 versus Avastin RA; ^§^*p* < 0.05, ^§§^*p* < 0.01, ^§§§^*p* < 0.001 versus right untreated eye. RE: right eye; LE: left eye.

Digital images of the ADPase-stained retinal flatmounts take at 10x magnification were analyzed using WimRetina retinal vessel quantification image analysis software (Wimasis, Munich, Germany), as shown in Figure 1(a). The four quadrants of three retinas from each group (*n* = 12 measurements per group) were analyzed in a masked manner for vascular density (%, calculated by dividing the number of pixels of the vessels by the total number of pixels of the region of interest), total vascular area, number of branching points (where two or more segments converge), number of segments (number of individual vessel segments), and mean segment length.

### 2.8. Statistical Analysis

Data were analyzed using analyses of variance (ANOVA) for normally distributed data comparing differences among the oxygen groups (RA versus 50% O_2_ versus IH) within the saline- and Avastin-treated groups following Bartlett's test for equality of variances. For nonnormally distributed data, the Kruskal-Wallis test was used. Post hoc analysis was performed using the Tukey, Bonferoni, and Student-Newman-Keuls tests for significance. Unpaired *t*-tests were conducted to compare saline versus Avastin within each oxygen environment following Levene's test for equality of variances for normally distributed data. Mann–Whitney *U* test was used for nonnormally distributed data. Significance was set at *p* < 0.05, and data are reported as mean ± SD. All analyses were two-tailed and performed using SPSS version 20.0 (SPSS Inc., Chicago, IL).

## 3. Results

### 3.1. Avastin Decreases Somatic Growth in RA

Growth parameters are listed in Table 1. In RA, percentage change in somatic growth was significantly reduced with Avastin treatment at P23 and P45. Similar reductions in brain/body weight and lung/body weight ratios occurred at P45. Hyperoxia itself reduced somatic growth at P23 with minimal catch-up growth at P45. Avastin treatment in hyperoxia increased somatic growth although weight accretion did not quite achieve control levels. There were no significant effects of Avastin on brain/body or lung/body weight ratios in response to treatment in hyperoxia. IH caused similar reductions in somatic growth with minimal adjustments in response to Avastin treatment. Treatment with Avastin in IH increased brain/body and lung/body weight ratios at P23.

### 3.2. Avastin Increases VEGF in IH

**Figure 2:**
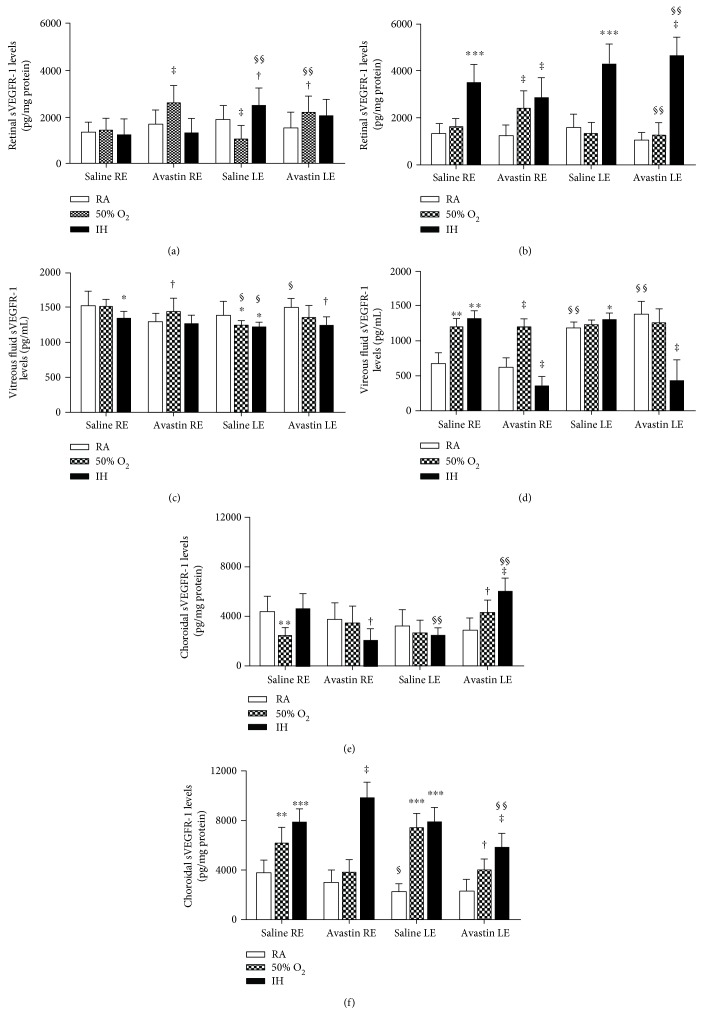
Effects of Avastin on retinal (a, b), vitreous fluid (c, d), and choroidal (e, f) sVEGFR-1 levels in adolescent 23-day-old (a, c, and e) and pubertal 45-day-old (b, d, and f) rats exposed to room air (RA), hyperoxia (50% O_2_), and intermittent hypoxia (IH). Groups are as described in Figure 1. Data are presented as mean ± SD (*n* = 6 samples/group). ^∗^*p* < 0.05, ^∗∗^*p* < 0.01, ^∗∗∗^*p* < 0.001 versus saline RA; ^†^*p* < 0.05, ^‡^*p* < 0.01 versus Avastin RA; ^§^*p* < 0.05, ^§§^*p* < 0.01, versus right untreated eye. RE: right eye; LE: left eye.

To establish whether Avastin has effects on systemic VEGF, we examined the levels in serum at P23 and P45 (Table 2). At P23, Avastin had no effect on serum VEGF although the levels were significantly suppressed with hyperoxia and IH. At P45, the levels of VEGF in the untreated animals exposed to hyperoxia and IH remained lower than those in the RA animals. Animals treated with Avastin in RA had lower VEGF levels compared to those treated with saline, while treatment in IH caused a robust increase at P45. To determine whether Avastin affects the untreated eyes, we examined VEGF levels in the retina, VF, and choroid (Figure 2). In the retina, Avastin caused similar elevations in VEGF levels in the right eye and in the left eyes exposed to hyperoxia and IH at P23 (Figure 2(a)). At P45, retinal VEGF levels remained substantially higher in the left and right eyes of all groups exposed to IH despite treatment (Figure 2(b)). At P23, there was a significant increase in VF VEGF levels in the right untreated eyes compared to RA (Figure 2(c)). At P45, Avastin increased VF VEGF levels in the left and right eyes exposed to IH (Figure 2(d)). In the choroid, Avastin increased VEGF levels in the right eyes exposed to IH and in the left eyes exposed to hyperoxia and IH (Figure 2(e)). At P45, choroidal VEGF remained elevated in the saline-treated right and left eyes exposed to hyperoxia and IH compared to RA controls, and in the Avatin-treated right eye exposed to IH (Figure 2(f)).

### 3.3. Avastin Decreases sVEGR-1 in IH

**Figure 3:**
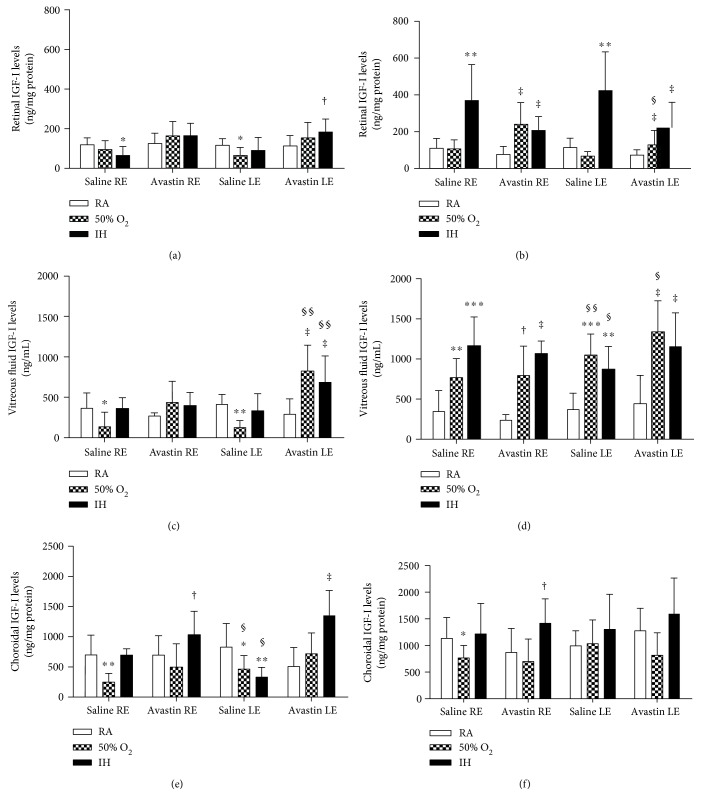
Effects of Avastin on retinal (a, b), vitreous fluid (c, d), and choroidal (e, f) IGF-1 levels in adolescent 23-day-old (a, c, and e) and pubertal 45-day-old (b, d, and f) rats exposed to room air (RA), hyperoxia (50% O_2_), and intermittent hypoxia (IH). Groups are as described in Figure 1. Data are presented as mean ± SD (*n* = 6 samples/group). ^∗^*p* < 0.05, ^∗∗^*p* < 0.01, ^∗∗∗^*p* < 0.001 versus saline RA; ^†^*p* < 0.05, ^‡^*p* < 0.01 versus Avastin RA; ^§^*p* < 0.05, ^§§^*p* < 0.01 versus right untreated eye. RE: right eye; LE: left eye.

sVEGFR-1 is an endogenous inhibitor of VEGF action. To establish whether Avastin influences systemic sVEGFR-1, we examined the levels in serum at P23 and P45 (Table 2). Avastin had no effect on serum sVEGFR-1 levels in any oxygen environment, although the levels were suppressed with hyperoxia and IH at P23 and sustained until P45. At P23, Avastin increased retinal sVEGFR-1 levels in the untreated right and treated left eyes exposed to hyperoxia (Figure 3(a)). At P45, all IH exposed eyes had higher sVEGFR-1 levels regardless of treatment (Figure 3(b)). At P23, Avastin suppressed VF sVEGFR-1 levels in the untreated right eye in RA and in the treated left eye in IH (Figure 3(c)). At P45, Avastin suppressed sVEGFR-1 levels in the VF of both untreated and treated eyes exposed to IH (Figure 3(d)). In the choroid, a different response was noted at P23. Avastin treatment suppressed sVEGFR-1 levels in the untreated right eyes exposed to IH but increased it in the treated eyes exposed to hyperoxia and IH (Figure 3(e)). At P45, choroidal sVEGFR-1 levels were elevated in the eyes exposed to hyperoxia and IH. Avastin treatment appeared to decrease the levels in the right untreated eyes exposed to hyperoxia and in the left-treated eyes exposed to hyperoxia and IH (Figure 3(f)).

### 3.4. Avastin Increases Retinal and Choroidal IGF-I in IH

**Figure 4:**
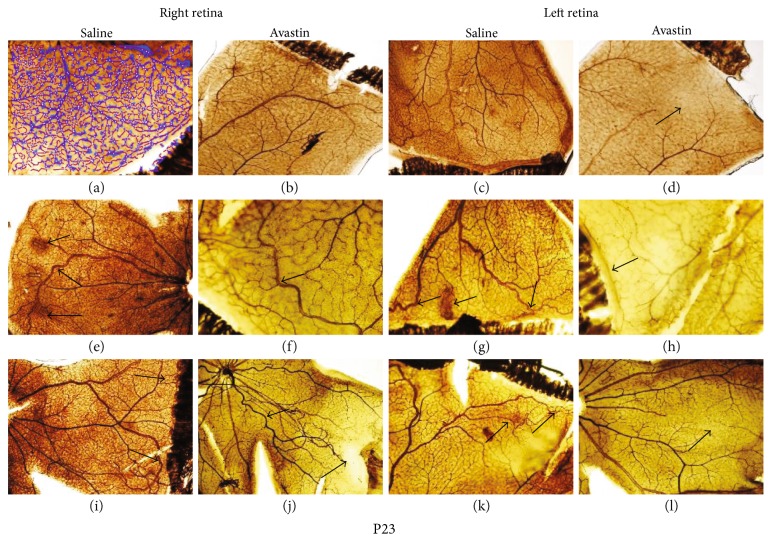
Retinal flatmounts showing ADPase-stained retinas from 23-day-old rat exposed to RA (a–d), 50% O2 (e–h), and IH (i–l). (a), (e), and (i) are right untreated retinas from saline-treated groups; (b), (f), and (j) are right untreated retinas from Avastin-treated groups; (c), (g), and (k) are saline-treated left retinas; and (d), (h), and (l) are Avastin-treated left retinas. (a) is a representative image of the WimRetina analysis for quantitation of vascular density parameters. Images are 10x magnification. Scale bar, 100 *μ*m.

IGF-I is a permissive factor for VEGF action. To establish whether Avastin influences systemic IGF-I, we examined the levels in serum at P23 and P45 (Table 2). Avastin suppressed serum IGF-I levels in IH at P23. However, at P45, both saline and Avastin-treated groups in IH had lower serum IGF-I levels. At P23, Avastin caused significant increases in retinal IGF-I levels particularly when administered in IH (Figure 4(a)). At P45, IH increased retinal IGF-I levels in the saline-treated eyes, while hyperoxia increased it in the Avastin-treated eyes (Figure 4(b)). At P23, Avastin increased VF IGF-I levels in the hyperoxia- and IH-exposed treated left eyes (Figure 4(c)). At P45, hyperoxia and IH caused elevations in VF IGF-I levels in all eyes (Figure 4(d)). Choroidal IGF-I levels were 3-4-fold higher than that of the retina. Avastin increased choroidal IGF-I levels when administered in IH at P23 (Figure 4(e)). Choroidal IGF-I levels remained elevated at P45 in all eyes exposed to IH (Figure 4(f)).

### 3.5. Avastin Causes Long-Term Retinal Neovascularization and Hemorrhage

**Figure 5:**
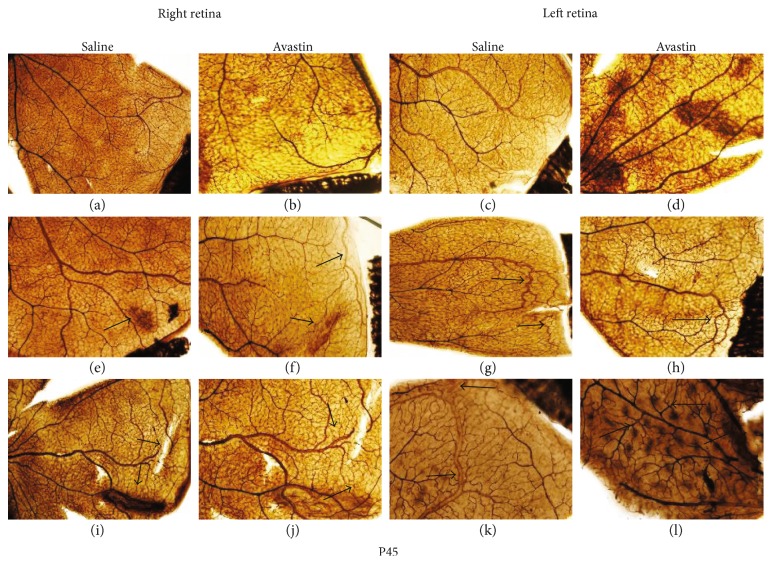
Retinal flatmounts showing ADPase-stained retinas from 45-day-old rats. Groups are as described in Figure 4. Images are 10x magnification. Scale bar, 100 *μ*m.

At P23, Avastin-treated left eyes in RA (Figure 1(d)), hyperoxia (Figure 1(h)), and IH (Figure 1(l)) had decreased branching elements and a more limited capillary plexus compared to saline control left eyes particularly those exposed to hyperoxia. There was a difference in retinal vasculature seen in the fellow eye as well, with the fellow right eye consistently showing greater amounts of neovascularization and anastomoses in RA (Figure 1(b)), vessel dilatation in hyperoxia (Figure 1(f)), and disorganized tortuous vessels, with decreased branching at the periphery in IH (Figure 1(j)). The untreated right and left eyes showed characteristics consistent with OIR following exposure to hyperoxia (Figures 1(e) and 1(g)) and IH (Figures 1(i) and 1(k)). At P45, retinal vessels of Avastin-treated left eyes were still prominent, but greater amounts of intraretinal hemorrhage were seen in RA (Figure 5(d)) and a greater degree in IH (Figure 5(l)). There was also a suppressive effect on the untreated fellow right eyes with decreased capillary networks in RA (Figure 5(b)) and in hyperoxia (Figure 5(f)), but in IH, there was evidence of dilated abundant vascular networks (Figure 5(j)). In the saline-treated eyes, characteristics consistent with OIR persisted at P45 in the hyperoxia (Figures 5(e) and 5(g)) and IH (Figures 5(i) and 5(k)) groups.

### 3.6. Avastin Causes Severe Retinal Abnormalities

**Figure 6:**
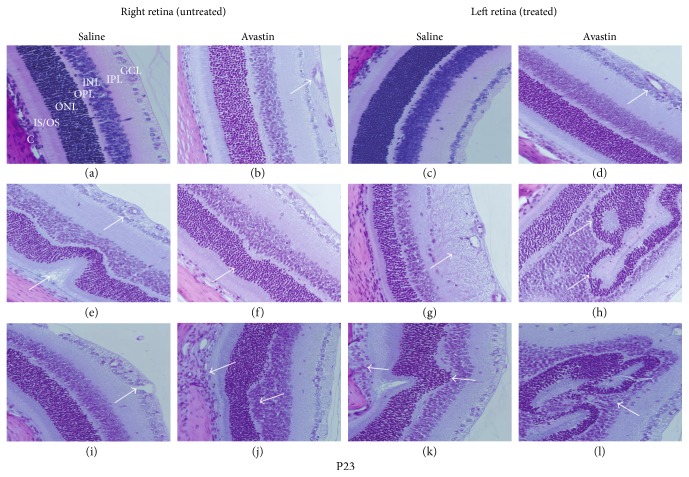
H&E stain of retinal layers from 23-day-old rat exposed to RA (a–d), 50% O_2_ (e–h), and IH (i–l). (a), (e), and (i) are right untreated retinas from saline-treated groups; (b), (f), and (j) are right untreated retinas from Avastin-treated groups; (c), (g), and (k) are saline-treated left retinas; and (d), (h), and (l) are Avastin-treated left retinas. Images are 40x magnification. Scale bar, 20 *μ*m.

**Figure 7:**
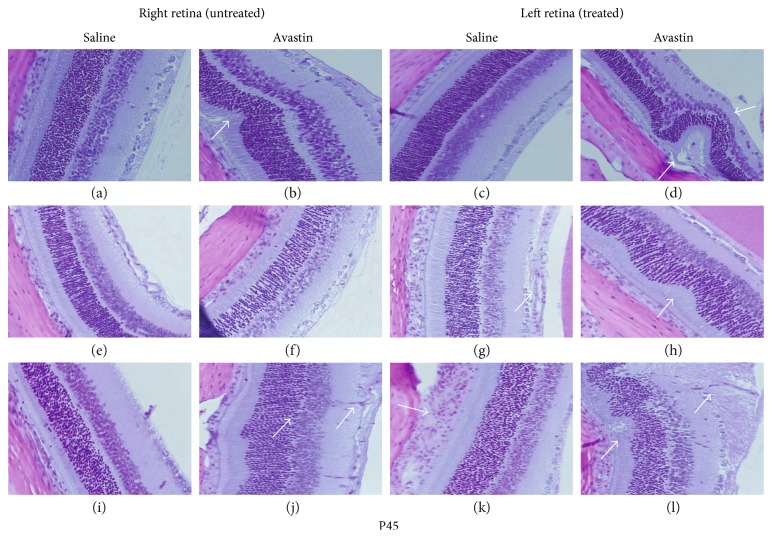
H&E stain of retinal layers from 45-day-old rats. Groups are as described in Figure 6. Images are 40x magnification. Scale bar, 20 *μ*m.

Figures 6 and 7 show the H&E-stained retinal layers from P23 and P45 rats, respectively. The layers are labeled in Figure 6(a): NFL (nerve fiber layer), GCL (ganglion cell layer), IPL (inner plexform layer), INL (inner nuclear layer), OPL (outer plexiform layer), ONL (outer nuclear layer), IS/OS (photoreceptor inner segment/outer segment laye), and C (choroid)). A–D represent the RA groups, E–H are the corresponding hyperoxia (50% O_2_) groups, and I–L are the corresponding IH (50% O_2_/12% O_2_) groups. All images were acquired at the central retina where most of the damage was located. At P23, Avastin treatment in RA caused NFL loss and increased blood vessel size (arrow) in the left retina (Figure 6(d)). Similar effects were noted in the untreated right retina (Figure 6(b), arrows). Avastin treatment in hyperoxia caused severe abnormalities in the IPL and INL (Figure 6(h), arrows). Saline treatment in the left eye caused significant NFL swelling and GCL degeneration (Figure 6(g), arrow). In the untreated right eye, there were mild retinal folds in the Avastin group (Figure 6(f), arrow), moderate retinal folds with photoreceptor loss, and increased blood vessel caliber and wall thickness (Figure 6(e), arrows). Severe abnormalities in the IPL and INL persisted with Avastin treatment in IH (Figure 6(l), arrow), while in the untreated eye, there was mild retinal folds and choroidal neovascularization (Figure 6(j), arrows). Saline treatment in IH caused retinal folds, photoreceptor loss, and choroidal neovascularization (Figure 6(k), arrow), while in the untreated right retina, there was mild NFL swelling and blood vessel dilatation (Figure 6(i), arrow). At P45, Avastin treatment in RA caused retinal folds (arrow), photoreceptor degeneration, and moderate loss of the NFL and GCL (arrows) in the left retina (Figure 7(d)). In the untreated right retina, Avastin caused mild retinal folds (Figure 7(b), arrows). Avastin treatment in hyperoxia caused mild retinal folds and loss or narrowing of the outer plexiform layer (Figure 7(h), arrows). Treatment with Avastin in IH caused retinal folds, moderate INL loss, and degeneration of NFL and GCL (Figure 7(l), arrows). Saline treatment in IH caused marked choroidal dilation (Figure 7(k), arrow). In the untreated right retina, Avastin caused mild retinal folds, loss of OPL, and GC atrophy (Figure 7(j), arrows).

### 3.7. Avastin Effects On Vascular Density

Quantification of vascular density for the right contralateral eyes (RE) and the left-treated eyes (LE) is presented in Tables 3 and 4, respectively. Data showed that exposure to 50% O_2_ and IH significantly reduced vascular density and branching points, and the effects worsened with Avastin treatment. The effects of Avastin treatment in IH persisted until P45.

## 4. Discussion

Whereas laser photocoagulation is the “gold standard” for the clinical treatment of ROP, there are instances when its use is associated with poor anatomic and visual outcomes. The use of pharmacotherapy is contemplated in these high-risk cases and is gaining popularity. Although safe and effective in adults with minimal systemic adverse outcomes, there is a concern that intravitreal exposure may have a far different safety profile in small neonates [15]. Although adverse events associated with Avastin in ROP are rare, they are not negligible and include retinal detachment, vitreous and retinal hemorrhage, choroidal rupture, and spreading via the bloodstream into the fellow eye [16–21, 36–39]. These adverse events suggest that the immature retina may be vulnerable to VEGF blockade which may cause breakdown of the blood-ocular barrier [40]. More importantly, if Avastin interferes with the immature blood-ocular barrier, it is possible that it may cross into the systemic circulation and have inhibitory effects on VEGF in the developing neonate's vital organs, such as the brain [21] and lungs [41]. There are no studies examining the effects of Avastin treatment in the setting of neonatal IH. This is crucial since preterm infants at risk for severe ROP experience several hundred episodes of IH over the first few weeks of life [3]. For this reason, we used a well-established model of OIR which closely resembles neonatal IH experienced by ELGANs [29–34]. An important finding that concurs with others is that Avastin leaks into the fellow untreated eye and the systemic circulation, providing evidence that the contralateral eye should never be used as a pure control eye if manipulations are performed in the study eye. Avastin has long-term adverse effects on the retina resulting in hemorrhage, neovascularization, and photoreceptor abnormalities, particularly when administered in IH. Given that Avastin is a permanent VEGF inhibitor and that VEGF is a vascular permeability factor, it is counter-intuitive that its use is associated with hemorrhage as reported in many human and animal studies including the present study. In addition, the manufacturer's package insert warns against the use of Avastin in patients with serious hemorrhage. Gastrointestinal bleeding, central nervous system (CNS) hemorrhage, and vaginal bleeding occurred up to 5-fold more frequently in patients receiving systemic Avastin. Serious or fatal pulmonary hemorrhage occurred in 31% of patients treated with Avastin (Avastin package insert). Our recent findings of pulmonary hemorrhage in rats treated with intravitreal Avastin concur with the package insert [41]. The robust rebound elevations in VEGF, including in the untreated fellow eye, may provide at least one mechanism. Thus, it is reasonable that preterm infants with chronic lung disease experiencing many episodes of IH will have elevated levels of VEGF via HIF_1*α*_ upregulation. VEGF, being a vascular permeability factor, will cause vascular leakiness and hemorrhage. This has been repeatedly shown in our rat model of OIR [29, 31–34]. Administration of intravitreal Avastin in the setting of IH will result in adverse ocular and systemic effects, due to drug leakage from damage vessels.

Serum VEGF levels are key indicators of the potential of intravitreal Avastin to cause systemic effects. Previous studies found decreased serum VEGF levels in rats that received murine VEGF-A antibody [16], as well as in human infants who received 0.25 or 0.5 mg intravitreal Avastin [42]. These studies suggest the ability of VEGF inhibitors to escape the vitreous and enter into the general circulation to cause considerable alterations in serum VEGF. No systemic sequelae were noted in these studies; however, both had relatively short study periods. In the present study, serum VEGF levels showed two new long-term patterns not previously seen. In rats raised in room air without OIR, intravitreal Avastin caused long-term anti-VEGF effects systemically as serum VEGF levels were significantly lower at P45. This resulted in a substantial difference in weight accretion which is most likely due to altered blood vascular and/or organ growth. This finding is troubling since VEGF is important for the development of the vital organs. In stark contrast to the rats raised in RA, the animals treated with intravitreal Avastin in IH showed a robust rebound elevation in systemic VEGF levels at P45, with no reciprocal body weight accretion. The accumulation of VEGF concurrent with IH-induced vascular impairment may lead to vessel permeability and hemorrhage. The increase in serum VEGF may represent a long-term compensatory systemic upregulation after the initial suppression by Avastin. It was interesting to note that Avastin had no substantial effect on serum sVEGFR-1, the endogenous inhibitor of VEGF which acts as a VEGF trap, or IGF-I which is a permissive factor for VEGF. sVEGFR-1 is associated with normalized angiogenesis [43]. However, Avastin treatment in IH caused a persistent decrease in serum sVEGFR-1 at P45. Similar latent systemic effects following intravitreal Avastin was demonstrated by Jalali et al. [19] who reported hepatic dysfunction in a 3-month old infant. Together, these findings confirm that Avastin enters general circulation and stays for weeks, which may result in permanent systemic adverse effects [42]. Therefore, treatment of preterm infants with already compromised blood-retinal barrier and retinal microvascular impairment will allow for even more drug to enter the systemic circulation, thus causing worse morbidities.

Long-term human infant studies of intravitreal Avastin are lacking. Martinez-Castellanos et al. [8] had the longest prospective case study of 5 years and actually used an adult dose of 1.25 mg Avastin per injection with encouraging results. All eyes in that study showed excellent anatomical success as well as visual outcomes; however, the study only included 13 patients and did not have a control group. In our study, the long-term effects of Avastin were most evident in the ocular compartment as evidenced by the retinal flatmounts and H&E stains. It should be noted that Avastin was administered on day 14, and samples were collected at day 23. Therefore, the increased retinal VEGF levels one week later may represent rebound effects. At P45, all IH exposed animals had higher retinal and choroidal VEGF levels, including the Avastin-treated groups. These high levels correlated with characteristics consistent with ROP (Figure 5) and are consonant with previous findings of a “late reactivation” phenomenon of ROP after intravitreal Avastin [17]. There are also case reports of choroidal ischemia with exudative retinal detachment, retinal break with macular hole, perivascular exudation and arterial narrowing with optic atrophy, and RPE/choroidal rupture [17–19]. A bilateral effect of unilateral Avastin has been previously reported [20]. Our data provide support to those previous findings, particularly when Avastin was administered in IH, which is most likely due to increased vessel permeability. The retinal folds and photoreceptor damage noted in the present study was also consistent with the previous reports [23].

One phenomenon that is often overlooked is the role of VEGF in the choroid and RPE. The use of VEGF inhibitors (particularly in the premature neonate) will alter choroidal blood flow [44] and result in apoptosis of the photoreceptors [45, 46]. Indeed, Avastin has been shown to cause inactivation of RPE cells, profound retinal dysfunction, and rapid and progressive dysfunction of cone receptors as determined by ERG [46]. The images of photoreceptor abnormalities (particularly in the intermittent hypoxia groups) as demonstrated in our H&E stains, as well as those of others [23], confirm the devastating effects of Avastin on the photoreceptors. There is evidence that intravitreal Avastin injection may be beneficial in ROP, especially in cases of posterior or severe disease. However, a strong case can be made regarding the potential adverse effects of leakage into the systemic circulation and possibly delayed, long-term adverse ocular and systemic effects, confirming that the drug is not restricted to the intended site. This fact alone should be a cause for concern and warrants the parsimonious use of anti-VEGF therapies in the setting of neonatal IH. As with all medical therapies, one must seriously consider the risk-benefit ratio when deciding on using intravitreal Avastin in the preterm neonate with an already compromised blood retina barrier and who experiences frequent IH episodes. We agree with Morin et al. [21] that more long-term follow-up studies are warranted, particularly when anti-VEGF therapies are used in the preterm infant.

## Figures and Tables

**Table 1:** Growth parameters.

	Room air (RA)		50% O_2_		IH (50% O_2_/12%O_2_ cycling)	
	Saline	Avastin	Saline	Avastin	Saline	Avastin
*P23*						
% change in body weight	745.7 ± 1.3	449.6 ± 16.9^§§^	308.7 ± 16.4^∗∗^	524.0 ± 28.8^§§^	644.9 ± 16.6^∗∗^	525.4 ± 22.2^§§^
% change in length	101.1 ± 1.1	81.8 ± 3.1^§§^	77.1 ± 2.8^∗∗^	91.1 ± 2.9^‡§§^	94.5 ± 2.4^∗∗^	92.6 ± 3.7^†^
Brain/body weight ratio	0.027 ± 0.0006	0.0092 ± 0.091	0.053 ± 0.003^∗∗^	0.04 ± 0.002^§§^	0.023 ± 0.0008	0.04 ± 0.0014^§§^
Lung/body weight ratio	0.0097 ± 0.0008	0.015 ± 0.002	0.021 ± 0.001^∗∗^	0.016 ± 0.002	0.009 ± 0.0003	0.02 ± 0.0014^‡^
*P45*						
% change in body weight	2886.6 ± 111.2	2328.4 ± 76.7^§§^	2373.1 ± 109.5^∗∗^	2677.7 ± 109.2^†^	2613.2 ± 84.6	2583.2 ± 81.2
% change in length	247.9 ± 4.3	187.8 ± 4.7^§§^	193.3 ± 3.5^∗∗^	205.9 ± 3.7^†§^	206.8 ± 4.2^∗∗^	193.7 ± 4.3
Brain/body weight ratio	0.041 ± 0.002	0.011 ± 0.003^§§^	0.01 ± 0.0003^∗∗^	0.01 ± 0.0003	0.009 ± 0.0003^∗∗^	0.0093 ± 0.004
Lung/body weight ratio	0.091 ± 0.005	0.008 ± 0.0006^§§^	0.0062 ± 0.0002^∗∗^	0.0065 ± 0.0003^†^	0.006 ± 0.0007^∗∗^	0.005 ± 0.0005^‡^

Data are mean ± SD (*n* = 18 per group; ^∗∗^*p* < 0.01 versus saline RA; ^†^*p* < 0.05 and ^‡^*p* < 0.001 versus Avastin RA; ^§^*p* < 0.05 and ^§§^*p* < 0.01 versus saline). IH: intermittent hypoxia.

**Table 2:** Serum levels of VEGF, sVEGFR-1, and IGF-I.

Growth factors	Room air (RA)		50% O_2_		IH (50% O_2_/12% O_2_ cycling)	
	Saline	Avastin	Saline	Avastin	Saline	Avastin
*P23*						
VEGF (pg/mL)	79.9 ± 17.6	82.9 ± 25.3	32.7 ± 27.2^∗∗^	19.7 ± 2.3^‡^	8.9 ± 10.8^∗∗^	1.01 ± 2.3^‡^
sVEGFR-1 (pg/mL)	716.8 ± 130.0	665.7 ± 285.9	594.6 ± 144.0^∗^	412.7 ± 51.7^‡§^	387.6 ± 99.7^∗∗^	350.3 ± 75.0^‡^
IGF-I (pg/mL)	4197.5 ± 33.0	4222.7 ± 99.3	4017.4 ± 152.2	4137 ± 95.2	4222.9 ± 86.7	3151.0 ± 51.8^‡§§^
*P45*						
VEGF (pg/mL)	73.3 ± 36.1	33.0 ± 25.2^§^	30.8 ± 2.4^∗∗^	17.3 ± 14.6	39.5 ± 20.1^∗∗^	98.5 ± 110.8^‡§§^
sVEGFR-1 (pg/mL)	671.8 ± 194.4	623.6 ± 166.4	518.6 ± 154.6^∗^	519.9 ± 106.4	304.3 ± 50.5^∗∗^	380.5 ± 124.4^‡^
IGF-I (pg/mL)	4044.3 ± 112.1	4018.6 ± 60.8	3985.9 ± 159.3	4036.0 ± 89.1	3214.0 ± 277.3^∗∗^	3144.3 ± 32.7^‡^

Data are mean ± SD; ^∗^*p* < 0.05 and ^∗∗^*p* < 0.01 versus saline RA; ^‡^*p* < 0.01 versus Avastin RA; ^§^*p* < 0.05 and ^§^^§^*p* < 0.01 versus saline (*n* = 10/group). IH: intermittent hypoxia.

**Table 3:** Quantitation of retinal vasculature (right eyes).

	Room air (RA)		50% O_2_		IH (50% O_2_/12% O_2_ cycling)	
	Saline	Avastin	Saline	Avastin	Saline	Avastin
*P23*						
Vascular density (%)	35.8 ± 3.6	34.1 ± 6.7^§§^	33.4 ± 2.1^∗∗^	31.4 ± 3.3	33.5 ± 4.7^∗∗^	25.6 ± 4.5
Total vessel network	23134 ± 7786	18391 ± 4537	15330± 1803^∗∗^	15020 ± 2031^†^	16141 ± 3644^∗∗^	12867 ± 2292^‡§§^
Branching points	522.5 ± 41.1	415.5 ± 49.4	316.5 ± 45.4^∗∗^	261.8 ± 59.4^‡§§^	343.0 ± 33.8^∗∗^	235.3 ± 63.1^‡§§^
Number of segments	904.0 ± 103.2	732.6 ± 127.2^§§^	580.8 ± 98.1^∗∗^	493.8 ± 99.7^‡§§^	619.5 ± 117.5^∗∗^	449.3 ± 109.8^‡§§^
Mean segment length	26.0 ± 2.8	29.0 ± 1.4^§§^	26.8 ± 1.7	30.8 ± 3.4^§§^	27.3 ± 5.3	29.0 ± 2.3
*P45*						
Vascular density (%)	31.3 ± 4.5	33.1 ± 1.8	36.0 ± 3.1^∗^	34.5 ± 2.3	36.9 ± 4.4^∗∗^	30.9 ± 4.2^§§^
Total vessel network	15764 ± 2492	16849 ± 3147	18180 ± 1929^∗^	18792 ± 2616	19177 ± 2281^∗∗^	15483 ± 2488^§§^
Branching points	320.7 ± 83.5	287.8 ± 58.6	401.3 ± 56.0^∗^	241.3 ± 65.2^§§^	451.8 ± 63.4^∗∗^	290.4 ± 68.9^§§^
Number of segments	599.3 ± 127.4	628.4 ± 127.6	718.3 ± 73.8^∗^	711.2 ± 105.0	784.0 ± 140.3^∗∗^	530.0 ± 123.8^§§^
Mean segment length	26.3 ± 1.2	31.0 ± 4.7^§§^	25.3 ± 1.5	26.7 ± 4.2^†^	25.5 ± 3.4	29.7 ± 2.7^§§^

Data are mean ± SD; ^∗^*p* < 0.05, ^∗∗^*p* < 0.01 versus saline RA; ^†^*p* < 0.05, ^‡^*p* < 0.01 versus Avastin RA; ^§§^*p* < 0.01 versus saline (*n* = 3 retinas/group; 12 measurements per group). IH: intermittent hypoxia.

**Table 4:** Quantitation of retinal vasculature (left eyes).

	Room Air (RA)		50% O_2_		IH (50% O_2_/12% O_2_ Cycling)	
	Saline	Avastin	Saline	Avastin	Saline	Avastin
*P23*						
Vascular density (%)	34.3 ± 3.2	32.5 ± 1.5	32.7 ± 5.1	30.4 ± 6.4	36.2 ± 4.3	31.4 ± 3.4^§§^
Total vascular area	14928 ± 1332	17045.0 ± 1981^§§^	15544± 4433	14654 ± 4274^†^	17963 ± 2611^∗∗^	14339± 2668^‡§§^
Number of branching points	278.0 ± 13.4	347.3 ± 76.0^§§^	289.2 ± 103.8	267.8 ± 97.6	407.7 ± 113.1^∗∗^	255.0 ± 71.2^†§§^
Number of segments	515.7 ± 23.3	622.3 ± 127.6^§§^	533.2 ± 177.8	512.4 ± 78.1	721.5 ± 178.5^∗∗^	487.8 ± 132.3^†§§^
Mean segment length	29.0 ± 1.4	27.7 ± 3.1	30.2 ± 4.5	29.8 ± 4.2	25.5 ± 2.7^∗^	30.0 ± 3.2^§§^
*P45*						
Vascular density (%)	34.5 ± 3.4	29.8 ± 4.5	37.9 ± 2.0	31.6 ± 3.3^§^	38.1 ± 8.1	24.1 ± 5.8^‡§§^
Total vessel network	17233 ± 2158	16076 ± 2604	22101 ± 5106^∗^	17434 ± 3190^§^	19773 ± 4041	12715 ± 2772^†§§^
Branching points	364 ± 76.3	277.0 ± 58.6^§§^	522.8 ± 124.8^∗∗^	347.3 ± 55.8^§§^	518.0 ± 112.1^∗∗^	198.3 ± 91.0^†§§^
Number of segments	653.0 ± 119.0	524.0 ± 118.7^§§^	896.0 ± 187.9^∗∗^	622.5 ± 85.1^§§^	876.8 ± 222.2^∗^	382.8 ± 137.9^†§§^
Mean segment length	26.8 ± 2.4	27.0 ± 2.1	24.5 ± 0.6	28.3 ± 3.3^‡§§^	24.5 ± 7	34.5 ± 4.2^‡§§^

Data are mean ± SD; ^∗^*p* < 0.05, ^∗∗^*p* < 0.01 versus saline RA; ^†^*p* < 0.01, ^‡^*p* < 0.01 versus Avastin RA; ^§^*p* < 0.05 and ^§§^*p* < 0.01 versus saline (*n* = 3 retinas/group; 12 measurements per group). IH: intermittent hypoxia.
